# How a laboratory-based antimicrobial resistance (AMR) regional surveillance system can address large-scale and local AMR epidemiology: the MICRO-BIO experience

**DOI:** 10.3389/fpubh.2024.1341482

**Published:** 2024-02-12

**Authors:** Agnese Comelli, Martina Zanforlini, Arianna Mazzone, Palmino Pedroni, Umberto De Castro, Simona Scarioni, Anna Carole D’Amelio, Giulia Renisi, Alessandra Bandera, Andrea Gori, Simone Schiatti, Danilo Cereda, the MICROBIO LR Group

**Affiliations:** ^1^Infectious Diseases Unit, Foundation IRCCS Ca’ Granda Ospedale Maggiore Policlinico, Milan, Italy; ^2^External Consultant, Lombardy Region, Milan, Italy; ^3^Regional Company for Innovation and Purchasing Aria S.p.A., Milan, Italy; ^4^Department of Biomedical Sciences for Health, School of Public Health, University of Milan, Milan, Italy; ^5^School of Public Health, Vita-Salute San Raffaele University, Milan, Italy; ^6^ASST Fatebenefratelli Sacco, Ospedale Luigi Sacco, Milan, Italy; ^7^General Directorate for Health, Lombardy Region, Milan, Italy

**Keywords:** AMR surveillance tool, high-priority pathogens, AMR surveillance, antimicrobial resistance, AMR monitoring

## Abstract

Antimicrobial resistance is a significant threat to public health, with Italy experiencing substantial challenges in terms of AMR rate, surveillance system and activities to combat AMR. In response, the MICRO-BIO project was initiated as part of the National Plan to Combat Antibiotic Resistance by Region Lombardy health department. It was launched in 2018 with the aim of creating a surveillance tool by integrating data on bacterial isolates from microbiology laboratories. The participating laboratories were directly involved in reviewing and addressing discrepancies in the transmission data quality assessment. Despite the disruptions caused by COVID-19, 30 out of 33 laboratories in the Lombardy Region were successfully integrated by October 2023, with 1,201,000 microbiological data collected in the first nine months of 2023. In 2022 the analysis yielded 15,037 blood culture results from 20 labs passing validation. Data regarding the antimicrobial resistance profile of high-priority pathogens was analyzed at regional and single-hospital levels. The MICRO-BIO project represents a significant step toward strengthening AMR surveillance in a highly populated region. As a multi-disciplinary tool encompassing the fields of public health and IT (information technology), this tool has the potential to inform regional and local AMR epidemiology.

## 1 Introduction

Antimicrobial resistance is a significant global public health concern. According to the 2019 report on global bacterial antimicrobial resistance (AMR), 4.95 million people who died in 2019 suffered from drug-resistant bacterial infections. Out of this number, 1.27 million deaths were directly attributed to resistant bacteria, which is approximately equal to the mortality rates caused by malaria and HIV (1).

In 2015, the World Health Assembly put into effect a worldwide strategy to tackle the issue of antimicrobial resistance, the Global Action Plan (2). It includes five key objectives: (i) raising awareness and understanding of AMR through effective communication, education, and training; (ii) strengthening knowledge and evidence through surveillance and research; (iii) decreasing the incidence of infection through adequate sanitation, hygiene, and prevention measures; (iv) improving the use of antimicrobials in human and animal health; (v) encouraging more significant and sustainable investments that take into account the needs of all countries (2).

The Assembly concluded with a resolution committing all UN member countries to develop national action plans (NAPs) for antimicrobial resistance (2).

Monitoring and evaluating interventions for antibiotic-resistant bacteria requires robust information on infection incidence, as outlined in the action plans’ model.

In the WHO European Region, Italy has one of the highest burdens of infections caused by antibiotic-resistant bacteria (3, 4).

The prevalence of methicillin-resistant *S. aureus* (MRSA) among invasive samples is around 30% and has remained stable in the last three years (5). There is a growing concern regarding the rise of vancomycin-resistant *E. faecium* (VRE) strains, which increased threefold from 2015 to 2022, and now account for 30% of all *E. faecium* isolates (5). Resistance to third-generation cephalosporins is seen in *E. coli* and *K. pneumoniae* at rates of around 25 and 55%, respectively (5). *Acinetobacter* spp. shows over 80% resistance to carbapenems. Nearly a quarter of *K. pneumoniae* and more than 10% of *Pseudomonas aeruginosa* also show carbapenem resistance (5).

Compared to the European (EU/EAA) mean, the resistance rate is almost 1.5–2 times higher for third-generation cephalosporins in *E. coli* and *K. pneumoniae*, for methicillin in *S. aureus*, and for vancomycin in *E. faecium*. Carbapenem resistance in *K. pneumoniae* and *Acinetobacter* spp. is almost 2.5–3 times higher than the EU/EAA mean (4).

In Italy, there is a lack of a coordinated surveillance system for antimicrobial resistance (AMR) rates. The few data that are sent to the Ministry of Health (MoH) and the European Centre for Disease Prevention and Control (ECDC) originate from only a small group of regions, resulting in limited coverage overall. According to the 2022 Antimicrobial Resistance Surveillance System of the Italian National Health Institute (AR-ISS) report, the national overall coverage was 61.7% (5). Out of the 21 regions, 12 had coverage below 70%, with most of them clustered in the central and northern regions (5). The uneven geographical coverage of AMR surveillance in the country may be probably attributed to the highly fragmented national health system, which relies heavily on regional health systems. Another reason could be the absence of a coordinated national plan to measure AMR before the development of the National Plan to Combat Antibiotic Resistance in 2017 (PNCAR) (6).

Furthermore, fully implemented antimicrobial stewardship (AMS) projects are rarely implemented, and the COVID-19 pandemic has disrupted the progress made in the past years (7).

In this context, in 2016, the ECDC visited Italy to assess the country’s situation regarding the prevention and control of AMR (8).

Observations from this visit confirmed that the AMR situation in Italian hospitals and regions posed a significant threat to public health. The report highlighted the high levels of antimicrobial resistance in the country, particularly among carbapenem-resistant bacteria like *Acinetobacter baumannii* (78.3% carbapenem resistance rate) and *Enterobacteriaceae* (33.5%), according to the 2015 ECDC report. These findings indicated Italy as one of the European Union countries with the highest levels of antimicrobial resistance (8). For this reason, ECDC requested Italy to improve surveillance and notification of AMR and to monitor key indicators to efficaciously prevent and control AMR spread.

The ECDC country visit report also revealed an unequal distribution of laboratories participating in the national surveillance system (AR-ISS), mostly located in the northern regions, as already mentioned above.

The recommendations from the report compiled by ECDC led to the development of the National Plan to Combat Antibiotic Resistance (PNCAR), signed by the Italian MoH in December 2017 (6).

The PNCAR main objectives included AMR monitoring in human and animal health, healthcare-associated infections (HAI) surveillance and control of antibiotic consumption in human and veterinarian settings (6). Moreover, one of the early objectives was to extend national coverage (6).

In this paper, we describe the development of a laboratory-based AMR surveillance tool (MICRO-BIO) in an Italian region as a result of the 2017 PNCAR. Additionally, this paper provides an overview of the first experience reporting quantitative and qualitative data on bloodstream infections collected through MICRO-BIO tool. This is done to demonstrate possible application of the tool.

## 2 Context

The MICRO-BIO project started in 2016 as an initiative by the Region Lombardy (LR) health department following the ECDC’s country visit and stimulated by the national efforts to develop a NAP (PNCAR), which was signed successively in December 2017.

LR is the most populated region in Italy, with a resident population of around 11 million people, a territory of 23,861 square kilometers and 1,544 municipalities distributed in 12 provinces.

Although LR is located in the northern part of Italy, which is known to participate more actively in the AR-ISS, the data about LR’s participation has been unsatisfactory. In the first AR-ISS report of 2018, LR’s coverage in AR-ISS data was only 17.5%, while the national coverage was 35.8% (9). Since then, the figures have only increased to 56% in 2022, which is still lower than the national coverage overall (61.7%) and the surrounding regions that have an average coverage of 80% (5).

Therefore, the aim of the project was to fulfil the national and ECDC request to improve AMR surveillance and identify essential prevention and control indicators.

To ensure maximum participation, the Italian MoH made available a specific fund available to all healthcare facilities to share after integration.

The actions described above are legislated in two Regional Council Resolutions: Resolution X/7468 of 4/12/2017 and Resolution X/7630 of 28/12/2017.

## 3 Details

### 3.1 The MICRO-BIO surveillance tool

**Figure 1:**
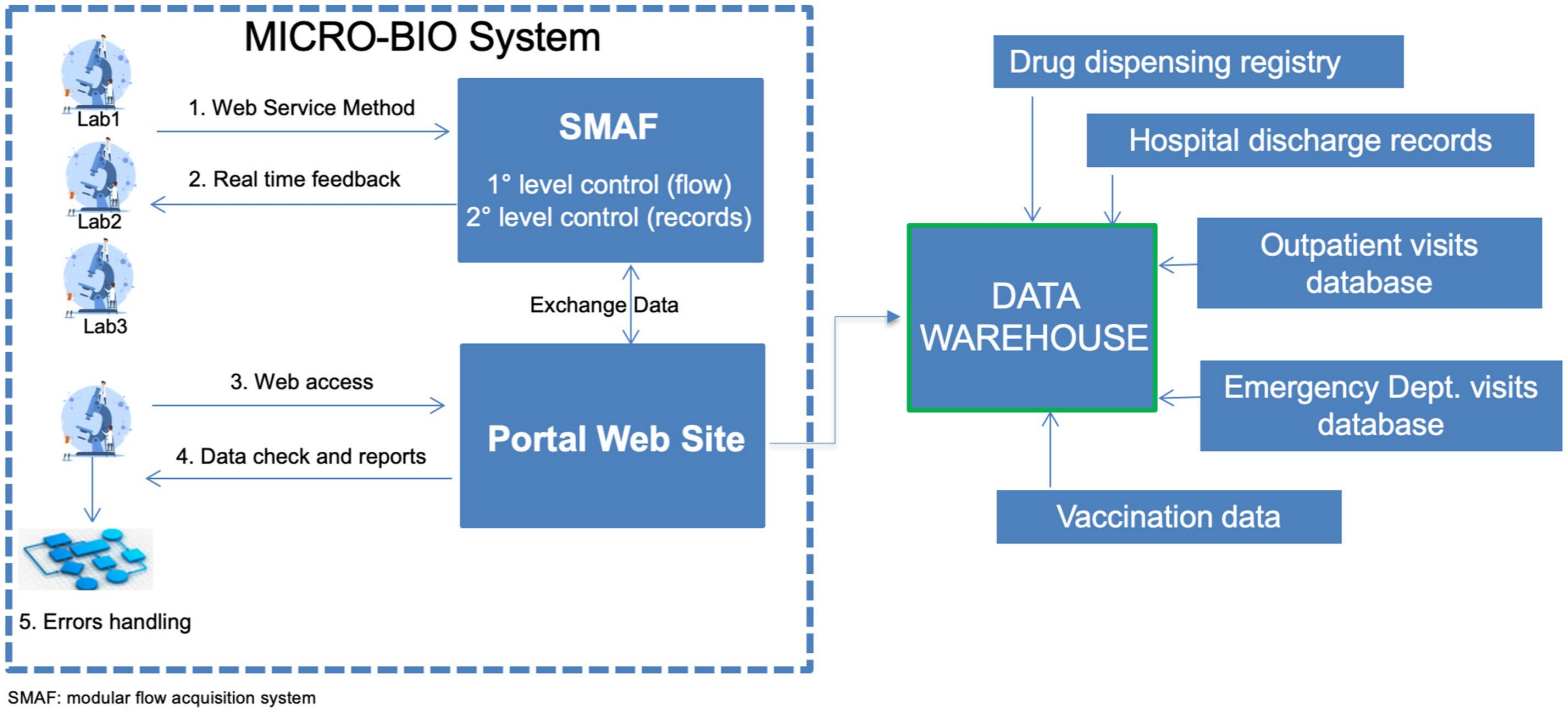
Schematic figure on the functioning of the MICRO-BIO surveillance system. Laboratories send their records through a dedicated web service on a daily to weekly basis. Those records are hosted by a modular flow acquisition system (SMAF) where two levels of data control are established. The first level of control includes automatic verification of the data format transmitted for each record: date, number, text formats and length of identification codes. After completing the first-level check, a second-level control was conducted to ensure that all mandatory fields were filled in. Then, real-time feedback was sent to the laboratory. SMAF exchanges data with the portal website that can be accessed by the participating laboratories. There reports are published, and queries on data checks are posted, allowing laboratories to handle possible errors. The portal website sends weekly all records to the data warehouse where microbiological data may be merged with the other health databases for specific purposes.

The MICRO-BIO surveillance tool integrates data from public microbiology labs of the LR via a regional service to the MICRO-BIO server. A schematic diagram of the functioning of the MICRO-BIO surveillance tool is provided in Figure 1, with details on each step.

This data flow was created through a collaborative effort between the microbiologists and the IT office of each healthcare facility, the private healthcare software companies that are responsible for managing hospital data, and the Regional Board on AMR control.

This Regional Board consists of the Director of the Prevention Unit at LR, members of the regional in-house company that acts as an interface for LR central authority and all public entities, as well as IT professionals and consultants specialized in microbiology and infectious diseases.

The tool was designed specifically for managing microbiological data related to bacteriology. Negative and positive results (the bacterial isolate and its attributes) were registered.

Samples collected in hospital wards and outpatient clinics for adults and pediatric patients are included. In order to create alerts, each microbiological data is associated with the name and date of collection for the health facility where the sample was taken. Table 1 lists the variables associated with each record.

**Table 1:** MICRO-BIO surveillance tool variables.

Variables description
Type of record (new, update or delete)^#^
Lab identification code^#^
Site of sample collection^#^ Hospital admission Outpatient clinic
Sample registration, date^#^
Sample collection, date and hour
Hospital admission, date and hour
ER access, date and hour
Hospital identification code^#^
Identification of prescriber^#^
Sample origin ward^#^
Identification of patient^#^
Birth date^#^
Sex^#^
City of residence^#^
Identification of the hospital admission (patient-hospital specific)^#^
Sample^#^ Blood Cerebral spinal fluid (CSF) Urine Upper respiratory way secretion Lower respiratory way secretion Pus Feces Prostheses Swab Other
Surveillance purpose^#^ Yes No
Test output^#^ Positive Negative
Germ identification^#^
Type of exam^#^ bacterial culture antigen detection
Antibiotic tested^#^
Antibiotic Susceptibility^#^ Susceptible Intermediate Resistant
MIC (minimum inhibitory concentration)
^a^If an antibiogram has been performed, this section will be repeated for each antibiotic that was tested. ^#^Mandatory information

Notably, as shown in Figure 1, laboratories send their records through a dedicated web service on a daily to weekly basis.

The MICRO-BIO portal facilitated the sharing of microbiological data with local health authorities, hospital directors, clinical microbiologists, infectious disease specialists, physicians managing patients with infections, and other stakeholders.

The MICRO-BIO data output allowed participation in the AR-ISS, and the data collected through this national system was then sent to the EARS-Net, the ECDC AMR surveillance network.

Moreover, it was possible to merge microbiological data with other databases such as Hospital Discharge Records, drug dispensing registry and ER diagnosis databases (see Figure 1). This helps achieve additional objectives: (i) combating sepsis campaign by linking microbiological data to the ANGUS-AHR algorithm that leverages the ICD9/ICD10 codes (10); (ii) surveillance of HAI; (iii) monitoring antimicrobial resistance through the processing of trend graphs, maps, and indicators and (iv) linking the antibiotic consumption according to the AWARE classification to antibiotic-resistant germs circulation.

### 3.2 Transmission data quality validation

Data quality reflects the completeness and validity of the data within the public health surveillance system (11).

The first step was to validate the proper integration of information systems. This ensured that the software used for data management in various healthcare facilities could effectively send information to MICRO-BIO.

Data completeness was ensured by requiring mandatory fields in the surveillance dataset form. If any selected information was missing, the whole record was prevented from being sent.

Mandatory fields are shown in Table 1. Figure 1 shows this validation step.

Data validity was performed once, at the moment each laboratory entered the surveillance system.

Each sending laboratory received a raw report of their transmitted data, and they were asked to revise it to evaluate if data reflected their internal figures in terms of data volume, relative proportion of cultural samples and sample distribution (e.g., if all wards were represented). They were asked, at the same time, to address any discrepancies that might have arisen due to incorrect submissions.

In a small context, such as a single region, the method adopted has allowed for the direct involvement and commitment of individual centers to an activity that was completed voluntarily.

However, data validity was not assessed by a rigorous study aimed to measure the sensitivity and positive prediction value of the surveillance system.

## 4 Results

### 4.1 The dynamic of laboratories’ integration

**Figure 2:**
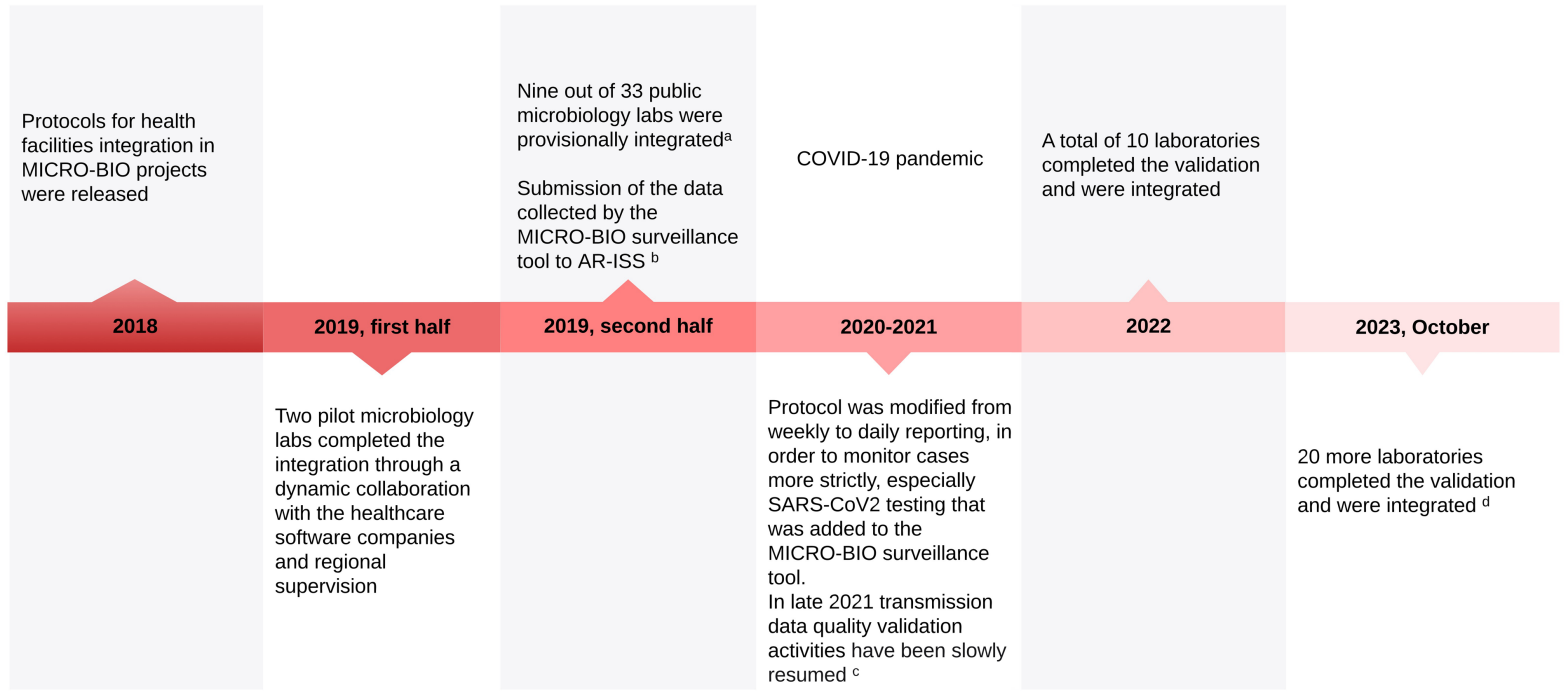
MICRO-BIO project implementation timeline. ^a^At this stage, the only requirement was the ability of the healthcare software house to send data to MICRO-BIO. No quality assessment was carried out. ^b^It was previously done independently by a few selected healthcare facilities. Sending data to AR-ISS by the MICRO-BIO database significantly increased the amount of data sent from LR to the Italian National Health Institute, improving adherence to ECDC standards. ^c^Regarding its main objectives, the MICRO-BIO project was delayed by the COVID-19 pandemic, and the activities for the transmission data quality validation phase slowly began in 2021 to be firmly resumed in 2022, thanks to the reduction of the pandemic pressure on the Region’s workload. ^d^The three remaining labs are expected to complete their integration by 2024.

The timeline of the implementation of the MICRO-BIO project is represented graphically in Figure 2.

**Figure 3:**
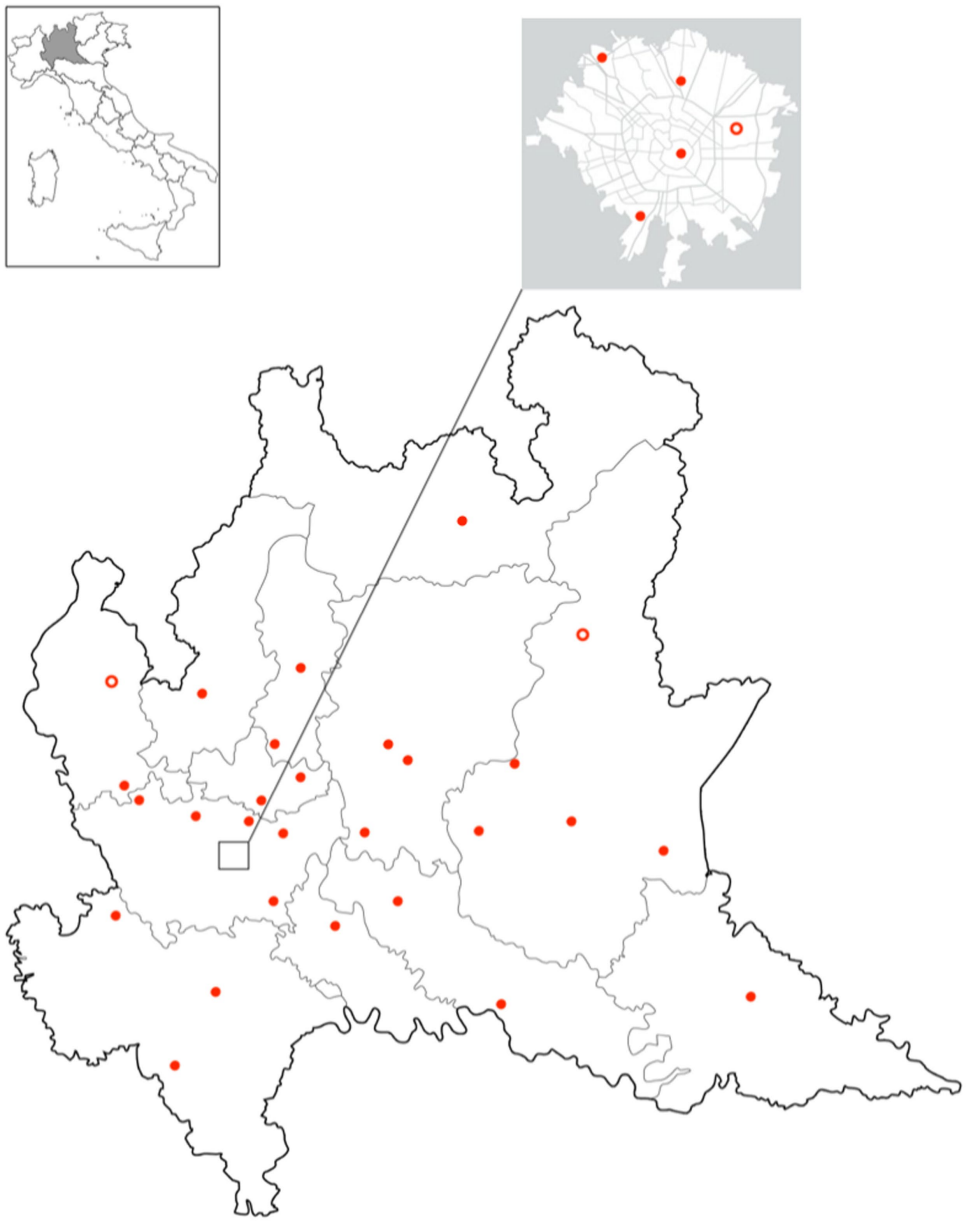
Geographical distribution of in Lombardy region. Full dots represent integrated laboratories and empty dots laboratories expected to be integrated in 2024.

As of October 2023, 30 out of 33 public laboratories in LR are integrated and regularly send microbiological data to the MICROBIO database. In Figure 3, the geographical distribution of integrated laboratories is shown.

In the first nine months of 2023, 1,201,000 microbiological data were sent by the integrated laboratories, including all types of samples and both positive and negative results.

After integrating the MICRO-BIO tool in nearly all public microbiology laboratories, the Regional Board appointed for AMR control decided to proceed with the first advanced epidemiologic measurement using 2022 data, as presented below.

Moreover, in late 2022, a team of experts consisting of infectious disease specialists, microbiologists, public health professionals, IT experts, and representatives of the LR regulatory bodies started working on the development of a new version of MICRO-BIO flow. The updated version is scheduled to be released in 2024 and will add the following variables: resistance mechanisms like extended-spectrum beta-lactamases and carbapenemases, testing for *Clostridioides difficile*, and molecular methods of bacterial detection.

Notably, if data is currently sent daily or weekly, data will be collected real-time in the 2024 release.

### 4.2 Application of the MICRO-BIO tool: bloodstream infections report

MICRO-BIO tool allows the collection and analysis of important epidemiological data such as bloodstream infections (BSI), where antimicrobial resistance has the most severe consequences. Beyond the epidemiologic purpose, the timely prescription of appropriate empiric antimicrobial therapy based on local epidemiology is crucial in reducing the clinical impact of these infections.

The eight priority pathogens defined by ECDC have been studied: *S. aureus, S. pneumoniae, E. faecalis, E. faecium, E. coli, K. pneumoniae, P. aeruginosa, A. baumannii* (4).

The impact of *Candida* spp. has been analyzed in light of their high mortality rates and the extensive use of antifungal drugs in several settings, especially azoles.

All microbiological results related to blood cultures from hospital wards, intensive care units, and the emergency department for adult patients were included in the analysis. Data from outpatient samples, patients aged 14 years or younger, and samples processed by laboratories outside the healthcare facilities of interest were excluded.

Several indicators were calculated: (i) the distribution of bacterial isolates causing BSI across the overall population, low and high intensive care units, age, and sex; (ii) cumulative antibiogram (with both percentage of resistance and 95% confidence intervals) for the eight priority pathogens and *Candida* spp., according to both the single drugs and the antibiotic classes. The cumulative antibiogram representation included the same information (percentage of resistance and 95% confidence intervals) extracted from the national AR-ISS (2021 data) (12) and ECDC reports (2020 data) (4) as comparators.

The epidemiological report was created for the LR as a whole and for the individual healthcare facilities whose labs participate in the MICRO-BIO project.

Moreover, the reports created for each single healthcare facility had a section specifically covering qualitative indicators. Specifically, proportions of positive and contaminated blood cultures were reported. Furthermore, the study investigated the adherence to the collection of a correct number of blood culture bottles (2 sets, 2 bottles each). In the output, it was possible to observe the percentage of BCE composed of a specific number of bottles (from 1 to N bottles) and compare those values among the different hospital departments. Indeed, performing less than four bottles per BCE deviates from correct clinical practice and significantly reduces exam sensitivity (13).

The LR report included 15,037 BSI from twenty public microbiology labs that passed the validation phase. It provided meaningful data on AMR prevalence in the region and highlighted some significant differences with the national (i.e., AR-ISS) figures.

These findings were essential to inform the AMR strategy of the region and to highlight different local needs compared to national guidelines.

The single healthcare facility report had a dual purpose. It provided valuable epidemiological data to inform infection and prevention control (IPC) priorities, as well as to help in the development of internal guidelines for empiric treatment of BSI.

Secondly, the report also used qualitative indicators to identify areas for audits and education activities. The analysis reveals that there are significant differences in the hospital wards in terms of collecting the correct number of blood culture bottles. Ideally, single blood culture sets should constitute less than 5% of the total (14).

Before the report’s release, a special event was held to present its objectives and explain the methods to different stakeholders, such as those in the healthcare sector and regional regulatory bodies. Subsequently, the report was sent to the relevant personnel of each participating hospital, including the hospital director, which contained both the LR report and the specific single-center report.

## 5 Discussion

Antimicrobial resistance is a global crisis, and some countries, including Italy, are experiencing its severe effects.

In this paper, we presented the launch, the goals and the challenges of an AMR surveillance tool in Lombardy, a highly populated region of Italy.

The measurement of AMR figures is crucial in the fight against antimicrobial resistance. The Global Antimicrobial Resistance and Use Surveillance System (GLASS), promoted by WHO, strongly supports this effort (15). Several examples in the literature demonstrate that countries from any level of income are investing resources in setting up AMR surveillance systems (16–19).

Data from AMR surveillance systems are essential for determining baseline AMR prevalence, setting priorities, and measuring intervention effectiveness.

Even if at a small scale, such data can inform local practices to promote valuable interventions that improve individual patient outcomes.

The MICRO-BIO project contribution is meaningful when considering local healthcare facilities as the main stakeholders: microbiology labs, local health authorities, and hospital stakeholders such as the hospital and medical directors. It allows them to analyze their data and use it to inform the hospital’s IPC and antimicrobial stewardship committee about which germs to prioritize and how to create an internal guideline for empiric treatment.

All public regional laboratories are served by the same few private healthcare software companies that applied a standardized model to integrate each laboratory into the regional database. This has allowed for the inclusion of small laboratories that took advantage of the upstream works done with the larger laboratories and, therefore, increased the regional coverage. Moreover, it allowed the monitoring of AMR in smaller facilities, where AMS projects and educational initiatives are seldom implemented.

As demonstrated through the BSI reports, the data collected are suitable for producing not only quantitative records but also qualitative indicators of blood culture collection.

This was an example that demonstrated how the MICRO-BIO tool is useful for analyzing the local context and improving the internal standardization of procedures through a targeted approach.

Moreover, it helps in monitoring adherence to regional and national objectives.

Since its birth, the MICRO-BIO project has highlighted that, despite the limitations posed by a parceled system when analyzing big data, the effort to improve homogeneity can result in a higher standard overall.

As a secondary effect, this project helped involve infectious disease specialists, microbiologists and hospital leaders in a regional network that will be valuable in managing possible regional events of interest, such as outbreaks or emerging pathogens detection.

The MICRO-BIO project presented several challenges and, consequently, some limitations.

The COVID-19 pandemic caused delays in the MICRO-BIO project roadmap. However, it also has caused some positive effects, emphasizing to health professionals and public opinion the importance of coordinated activities and central surveillance in managing health crises.

In this way, the COVID-19 pandemic was used as an opportunity to strengthen the collaboration between multiple healthcare professionals and receive support from regional stakeholders.

Notably, the MICRO-BIO project has allowed the uncovering of several limitations in the current practice.

Currently, there is neither a standardized vocabulary for microbiology results across different laboratories nor a unique guideline for microbiological reports in terms of antibiotics tested. Therefore, a shared reporting system would be beneficial for both epidemiological and clinical purposes.

Finally, there is no automatic system to detect and signal typing or possible laboratory errors and important results that may require urgent management. This limits the potential application of this surveillance system.

Data quality was not assessed by a rigorous methodology, only relying on single centers’ internal validation of the data transmitted to the MICRO-BIO data. As a consequence, we cannot present the sensitivity of this surveillance system and quality assessment is an imperative step to engage in the near future.

The MICRO-BIO project holds several potentials.

Currently, the project is expanding to include the remaining public and private laboratories. Indeed, in LR, a considerable percentage of healthcare facilities, including microbiology labs, belong to private organizations. Considering the movement of patients between private and public healthcare facilities, LR currently prioritizes the inclusion of private labs in the MICRO-BIO surveillance tool in order to involve these institutions in national and regional plans to contrast bacterial resistance spread.

The LR, the Italian National Health Institute and WHONET are collaborating to apply WHONET-SaTScan software (20, 21) to implement through MICRO-BIO an alert system to identify promptly cluster and outbreak identification and inform stakeholders in hospitals and public health public agencies.

Furthermore, the MICRO-BIO database can be linked to other regional information databases.

Merging the AMR prevalence data with the drug dispensing registry can reveal inconsistencies that require attention. For instance, a high prescribing rate of carbapenems may not be justified if *Enterobacteriaceae* susceptibility to narrow-spectrum antibiotics (e.g., cephalosporins and β-Lactams/β-Lactamase inhibitors) is preserved.

A common issue in preventing and controlling notifiable diseases is underreporting by physicians.

The MICRO-BIO surveillance tool can extract microbiological results that require notification (e.g., positive culture for *S. pneumoniae* in cerebro-spinal fluid). By comparing this data with the actual notification lists, it is possible to measure the underreporting rate and identify critical areas that may require an audit intervention. Even when the clinical suspicion is notified, the centralized flow of microbiological data can facilitate the work of the public health officer entitled to the case investigation.

Data told us that HAI severely affected hospital care both in high-income countries and low-and middle-income countries, becoming one of the main drivers of global morbidity and mortality (22).

Monitoring HAI incidence is crucial for allocating resources and measuring intervention efficacy, but it is a challenging task as it is generally a clinical diagnosis.

Merging Hospital Discharge Records, drug dispensing registry, ER diagnosis databases and MICRO-BIO database in an appropriate model may facilitate the identification and tracking of HAI. Some similar experiences have already been published with encouraging results (23, 24).

The updated version of PNCAR, released in 2022, promotes a One Health approach to addressing the challenge of antimicrobial resistance control (25). Indeed, AMR impact on humans is strictly related to its spread in the veterinarian setting and in the environment (26).

Luckily, the MICRO-BIO surveillance tool can host various data sources, including veterinary microbiology data and wastewater data related to contamination by antimicrobial-resistant germs.

This tool’s ability will enable effective responses to national and international objectives from one health perspective.

To conclude, this surveillance system can serve as a model for other regions in Italy where surveillance is not carried out through a systematic data flow but with dedicated queries to the larger laboratories requested by the regional health department or by timely surveys. Regional priorities and healthcare system organization may vary between regions. However, most public hospitals in Italy are served by the same few private healthcare software companies that work in LR. Therefore, the transmission system and the upstream data management to transform local laboratory data into a readable shape for the regional acquisition system can be efficiently shared within different regions, facilitating the surveillance implementation.

The potential result is the creation of a similar model among Italian regions. This model may enable the national MoH to analyze national data, inform international databases (e.g., ECDC, GLASS), compare AMR rates among regions, set regional objectives based on measured figures, and allocate resources efficiently.

## 6 Conclusion

In conclusion, the MICRO-BIO project, as a laboratory-based AMR regional surveillance system, can help address large-scale AMR epidemiology and management of antimicrobial resistance in local clinics. Measurement of its achievement in the next years would be critical to update and adapt the surveillance tool according to the regional needs and future epidemiological challenges.

## Data availability statement

The original contributions presented in the study are included in the article, further inquiries can be directed to the corresponding author.

## Author contributions

AC: Conceptualization, Methodology, Writing – original draft. MZ: Data curation, Formal analysis, Methodology, Writing – original draft. AM: Data curation, Formal analysis, Methodology, Writing – original draft. PP: Data curation, Methodology, Supervision, Writing – review & editing. UC: Data curation, Formal analysis, Writing – original draft. SSca: Writing – original draft. AD’A: Writing – original draft. GR: Validation, Writing – review & editing. AB: Supervision, Validation, Writing – review & editing. AG: Supervision, Validation, Writing – review & editing. SSch: Resources, Validation, Visualization, Writing – review & editing. DC: Resources, Supervision, Validation, Writing – review & editing.

## Group member of MICROBIO LR

Università degli Studi di Milano-Bicocca, Fondazione IRCCS San Gerardo dei Tintori – Monza, Monza, Italy: Paolo Bonfanti. Microbiology Unit, ASST Valle Olona, Italy: Gioconda Brigante. Directorate General for Health, Lombardy Region, Milan, Italy: Sabrina Buoro. Microbiology Unit, Fondazione IRCCS San Gerardo dei Tintori, Monza, Italy: Annalisa Cavallero. Clinical Microbiologists Italian Association (AMCLI) President, Italy: Pierangelo Clerici. Microbiology and Virology Laboratory, ASST “Papa Giovanni XXIII,” Bergamo, Italy: Claudio Farina. Department of Surgical and Morphological Sciences of Clinical Medicine, University of Insubria, Varese, Italy: Paolo Grossi. Clinical Microbiology and Virology Unit, “A. Manzoni” Hospital, Lecco, Italy: Francesco Luzzaro. Unit of Infectious Diseases, ASST Cremona, Cremona, Italy: Angelo Pan. Microbiological Analysis Unit, ASST Grande Ospedale Metropolitano Niguarda, Milan, Italy: Chiara Vismara. Intellera Consulting Spa: Sara Yacoub.
